# Ontogeny of sex differences in response to novel objects from adolescence to adulthood in lister-hooded rats

**DOI:** 10.1002/dev.20542

**Published:** 2011-03-31

**Authors:** De-Laine M Cyrenne, Gillian R Brown

**Affiliations:** School of Psychology, University of St AndrewsSt Andrews, UK E-mail: dc284@st-andrews.ac.uk

**Keywords:** novelty, sex difference, adolescence, rats, novel object recognition

## Abstract

In humans, novelty-seeking behavior peaks in adolescence and is higher in males than females. Relatively, little information is available regarding age and sex differences in response to novelty in rodents. In this study, male and female Lister-hooded rats were tested at early adolescence (postnatal day, pnd, 28), mid-adolescence (pnd 40), or early adulthood (pnd 80) in a novel object recognition task (*n* = 12 males/females per age group). Males displayed a higher preference for the novel object than females at mid-adolescence, with no sex difference at early adolescence. Adult females interacted with the novel object more than adult males, but not when side biases were removed. Sex differences at mid-adolescence were not found in other measures, suggesting that the difference at this age was specific to situations involving choice of novelty. The results are considered in the context of age- and sex-dependent interactions between gonadal hormones and the dopamine system. © 2011 Wiley Periodicals, Inc. Dev Psychobiol 53:670–676, 2011.

## INTRODUCTION

In human beings, adolescence can be associated with high levels of novelty- and sensation-seeking behavior (Arnett, 1992; Kelley, Schochet, & Landry, 2004; Zuckerman, 2006), and males are reported to engage in more sensation-seeking behavior than females across all age categories (Zuckerman, 2006). Attending to novelty during adolescence potentially allows maturing individuals to gain important information about the environment (Chambers, Taylor, & Potenza, 2003), while sex differences in novelty-seeking may result from sexual selection pressures favoring riskier strategies in males than females (Daly & Wilson, 1983; Spear, 2000). However, the biological mechanisms underlying age and sex differences in novelty-seeking are not well understood. The aim of this study was to examine age and sex differences in response to novelty in laboratory rats.

We used the novel object recognition (NOR) task (Berlyne, 1950; Ennaceur & Delacour, 1988), as this task forces rodents to confront novelty and also provides subjects with the opportunity to choose between a novel and a familiar stimulus. The procedure is to familiarize an animal to a novel arena, then place two objects into the arena and allow the animal to interact with the objects. During this first trial, Trial 1, the subject is “confronted” with novelty. One of these objects is then replaced with a completely novel item and, in Trial 2, the animal has the “choice” of interacting with the novel versus the familiar object. Rodents generally spend more time interacting with the novel than the familiar object in Trial 2 (Ennaceur & Delacour, 1988; Dere, Huston, & De Souza Silva, 2007).

The NOR task has been used extensively in rodent memory research; for instance, increasing the delay between the first and second trial to several hours has been shown to reduce the difference in response to the novel and familiar objects (Ennaceur & Delacour, 1988; Şik, van Nieuwehuyzen, Prickaerts, & Blokland, 2003). However, the NOR task also allows researchers to investigate the mechanisms involved in novelty preference (Besheer, Short, & Bevins, 2001). Lesions to the mesolimbic dopaminergic system influence NOR task performance, although the effects pharmacological manipulations of the dopamine system are less consistent (Dere et al., 2007; Hughes, 2007; Woolley, Marsden, Sleight, & Fone, 2003). Using a variant of the task with a short interval between the two trials (e.g., 2 min) reduces the probability that age or sex differences in response to the novel versus familiar object will result from differences in memory ability.

Only three studies have previously investigated age differences in NOR task performance using short inter-trial intervals in rodents and have produced inconsistent results: two studies on mice reported that the strength of preference for the novel object in the choice trial peaks at adolescence (Calamandrei, Rufini, Valanzano, & Puopolo, 2002; Ricceri, Colozza, & Calamandrei, 2000), while a study of male rats reported no difference in the strength of preference for the novel object during Trial 2 between adolescents and adults (Reger, Hovda, & Giza, 2009). Similarly, studies of sex differences in NOR task performance have produced inconsistent results: adult male rats have been reported to spend a higher (Frick & Gresack, 2003; Kosten, Lee, & Kim, 2007) or a lower proportion of time (Ghi, Orsetti, Gamalero, & Ferretti, 1999; Sutcliffe, Marshall, & Neill, 2007) interacting with the novel object in Trial 2 than adult females.

In this study, we examined the performance of male and female Lister-hooded rats on the NOR task at early adolescence (postnatal day, pnd 28), mid-adolescence (pnd 40), or early adulthood (pnd 80; age categories are based on Tirelli, Laviola, & Adriani, 2003) using a 2-min inter-trial interval. The Lister-hooded rat is a pigmented, outbred strain that is widely used in cognitive and visual tasks in the UK and other parts of Europe (McDermott & Kelly, 2008). In addition to collecting data on interactions with the objects during Trials 1 and 2, we measured locomotor activity in the arena, as age and sex differences in locomotion could potentially influence object interactions.

## METHODS

### Subjects and Housing

The subjects were 36 male and 36 female Lister-hooded rats bred in-house (stock supplied by Harlan, UK). All animals were cage-housed (25 cm × 45 cm × 15 cm) with ad libitum access to soy-free rodent pellets and water. Housing rooms were controlled for temperature (20 ± 1°C) and humidity (55 ± 5%), and maintained on a 12-hr light:dark cycle (lights on 7 am). From pnd 17, pups were handled once per day and, at pnd 21, were weaned into same-sex sibling groups. At pnd 28, animals were housed as same-sex sibling pairs.

Each subject underwent behavioral testing only once, with different animals used in each age group. Subjects were tested at pnd 28 (*n* = 12 males, 12 females), pnd 40 (*n* = 12 males, 12 females), or pnd 80 (*n* = 12 males, 12 females). One additional female (pnd 40) that failed to reach criteria (Behavioral measurements and data analyses section) was excluded from the study. The subjects were taken from 19 litters, and littermates and cage-mates were distributed as evenly as possible among all the age groups. All appropriate guidelines and requirements were adhered to, as set out in the Principles of Laboratory Animal Care (NIH, Publication No. 85–23, revised 1985) and the UK Home Office Animals (Scientific Procedures) Act 1986.

### Apparatus and Experimental Design

The testing apparatus was a wooden, light grey-painted square chamber, measuring 67 cm × 67 cm × 45 cm (l × w × h), with a solid floor constructed of the same material. Three objects were used during the experiment (yellow rubber toy, glass jar filled with rocks, blue plastic bottle filled with sand) and were chosen to deter climbing and chewing. A pilot study with adult male and female rats showed that, from a range of objects, the amount of time spent interacting was very similar for these items. The apparatus was surrounded by a black curtain, and a video camera attached to the ceiling relayed images to a computer. All tests were conducted between 09:00 and 14:00 hr in the same testing room under dim, white light (approximately 25 lux), and a white noise generator was used to mask external sounds.

At the beginning of the test session, a subject was brought to the testing room in a carrying box (42 cm × 26 cm × 13 cm) and placed into the empty apparatus for a 10-min familiarization session. The animal was then returned to the carrying box for 2 min while the apparatus was cleaned with a 70% ethanol solution and allowed to dry. Two objects were placed into the apparatus in adjacent quadrants, 15 cm apart and 8 cm from the wall. The animal was placed into an empty quadrant, facing away from the objects, for a 5-min session, Trial 1, during which the subject had the opportunity to interact with the two objects. At the end of Trial 1, the animal was returned to the carrying box for an inter-trial interval of 2 min, during which one of the objects was replaced by a novel object. The apparatus and objects were cleaned as before, and the animal was reintroduced to the apparatus for another 5-min session, Trial 2. The object that remained from the first trial was considered the familiar object, and the new object was considered the novel object. At the end of Trial 2, the subject was returned to the home cage, and all objects and apparatus were cleaned again in preparation for the next subject. The objects used in each trial were counterbalanced across subjects and between age groups, and whether the left- or right-hand object was replaced in Trial 2 was also counterbalanced.

### Behavioral Measurements and Data Analyses

All sessions were recorded directly onto the computer. Measures of object interaction were recorded manually using in-house software, while locomotor activity was analysed using EthoVision XT 5.0 software (Noldus Information Technology, Wageningen, The Netherlands, 2008).

Behavioral measures collected during Trials 1 and 2 included the *amount of time spent moving* and the *amount of time spent interacting with each object*. Object interaction was defined as the nose being in contact with an object, which excluded behaviors such as backing into an object, tail only contact, or time resting next to an object. Any animal that did not exhibit a minimum of 5 s of total contact with the objects in Trial 1 and at least 1 s contact with either object in Trials 1 and 2 was excluded from the study (one female at pnd 40). These criteria are comparable to those used in previous NOR studies (e.g., Anderson et al., 2004).

Two measures of novelty preference were calculated. The first measure, referred to as *preference for novelty*, was calculated as the proportion of time spent interacting with the novel versus the familiar object in Trial 2, converted to a percentage [(Time with novel − Time with familiar)/(Time with novel + Time with familiar) × 100]. A positive value indicates a preference for the novel object, while a negative value indicates a preference for the familiar object, and a score of zero indicates equal preference for the two objects.

The second measure, referred to as *preference change*, takes into account any initial biases by comparing the proportion of time spent with the two objects in Trial 1 to the proportion of time spent with the two objects in Trial 2. Previous research has reported that individual rats exhibit side-biases in behavioral tests and that rotational behavior differs between ages and sexes (e.g., Becker, Robinson, & Lorenz, 1982; Hyde & Jerussi, 1983; Schwarting & Borta, 2005). To take into account any biases that could affect the time spent with either object in Trial 1 (including individual preferences for a specific object), a side preference was calculated for both Trials 1 and 2 [(Time with right object − Time with left object)/(Total time with both objects) × 100], with a negative value representing a left-side preference, and a positive value indicating a right-side preference. *Preference change* was then calculated as the change in object contact times from Trial 1 (T1) to Trial 2 (T2), [(T2Right − T2Left)/(T2Right + T2Left)] × 100 − [(T1Right−T1Left)/(T1Right + T1Left)] × 100. The preference change value was adjusted to positive (+) if contact changed towards the novel object, or to negative (−) if contact changed towards the familiar object. Therefore, if the preference ratio increased between trials in the direction of the novel object, this score would have a positive value, and vice versa.

### Statistical Analyses

Repeated-measures analyses of variance (ANOVAs) were used to examine age and sex differences locomotor and object contact measures across the two trials. Correlations between novelty-preference scores and these other behavioral measures were examined (Pearson correlations), and analyses of co-variance (ANCOVAs) were subsequently used to examine whether preference scores in Trial 2 differed with sex and age. One-sample *t*-tests were used to examine whether groups of animals showed a significant preference for the novel object, as indicated by a score significantly greater than zero. Bonferroni pairwise comparisons were used to investigate age and sex differences. An *α* value of .05 was used throughout. Data were analyzed using SPSS 17.0 for Windows (2009). Effect size (partial-eta squared, 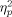) and power (*β*) values for ANOVAs were calculated by SPSS. Cohen's *d* and power for *t*-tests were calculated with G*Power Version 3.0.8.

## RESULTS

### Locomotion

**Figure 1:**
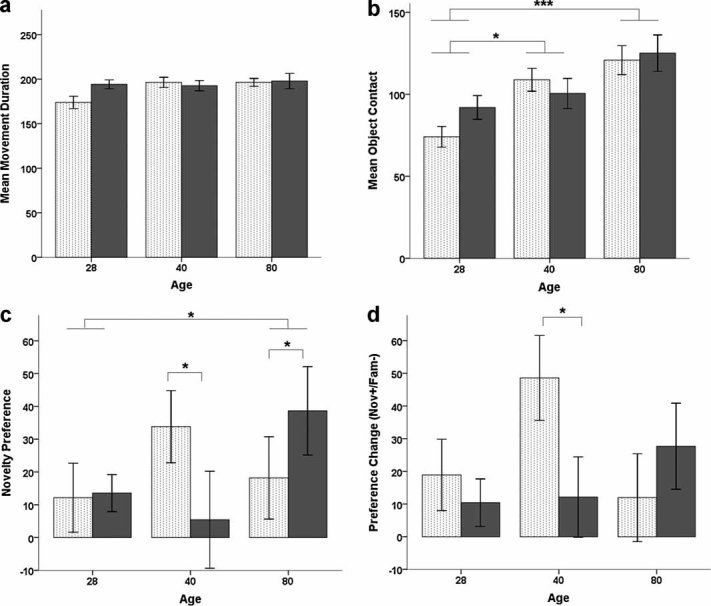
**a**: Amount of time spent moving (seconds) by age and sex for Trials 1 and 2 combined (means and SEMs). **b**: Total object contact (seconds) by age and sex across both Trials 1 and 2 (means and SEMs). **c**: Preference for novelty in Trial 2 by age and sex (means and SEMs). **d**: Preference change in Trial 2 by age and sex (means and SEMs). In all figures, stippled bars represent males, and grey bars represent females. Significant differences: *p < .05; **p < .01; ***p < .001.

The amount of time spent locomoting tended to increase with age (*F*_2,66_ = 2.49, *p* = .091, 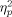 = .07, *β* = .48; pairwise comparisons non-significant, *p*s ≥ .116; Fig. 1a). There was no significant main effect of sex (*F*_1,66_ = 1.42, *p* = .238), nor were there significant interactions between sex and age, age and trial, or sex and trial (*F*s_1–2, 66_ ≤ 1.99, *p*s ≥ .134). Between the two trials, there was a significant decrease in movement from Trial 1 to Trial 2 (*F*_1,66_ = 15.40, *p* < .001, 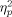 = .19, *β* = .97; Tab. 1).

**Table 1:** Means, in Seconds, and Standard Deviations of Behavioral Measures by Sex and Age Group (*n* = 12 per Group)

	Males			Females			Totals		
			
Age	28	40	80	28	40	80	28	40	80
Trial 1: Movement duration	187.25 (35.91)	204.06 (18.47)	206.43 (24.66)	206.67 (28.07)	202.51 (22.42)	193.51 (48.35)	196.96 (33.04)	203.29 (20.11)	199.97 (38.11)
Trial 2: Movement duration	160.53 (32.24)	188.66 (29.27)	186.43 (16.39)	181.78 (20.28)	182.86 (21.56)	202.27 (19.48)	171.15 (28.49)	185.76 (25.32)	194.35 (19.37)
Trial 1: Total object contact	78.60 (29.84)	120.89 (29.50)	115.13 (37.05)	103.93 (37.63)	102.26 (39.43)	126.06 (60.48)	91.26 (35.64)	111.57 (35.36)	120.60 (49.37)
Trial 2: Total object contact	69.51 (25.68)	96.78 (30.12)	126.83 (32.66)	79.00 (17.47)	98.64 (30.55)	124.05 (29.03)	74.69 (21.92)	97.71 (29.68)	125.27 (31.30)

*Note*. Numbers in parentheses are standard deviations.

### Total Amount of Contact With Objects

There was a significant main effect of age on the total amount of time spent in contact with the objects across both trials (*F*_2,66_ = 11.27, *p* < .001, 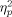 = .25, *β* = .99; Fig. 1b), with pairwise comparisons indicating increases from pnd 28 to 40 (*p* = .037) and pnd 28 to 80 (*p* < .001), but no difference between pnd 40 and 80 (*p* = .101). There was no significant main effect of sex (*F*_1,66_ = .44, *p* = .509), nor were there significant interactions between sex and age, age and trial, or sex and trial (*F*s_1–2, 66_ ≤ 2.21, *p*s ≥ .118). The total amount of time spent in contact with objects tended to decrease between Trial 1 and Trial 2 (*F*_1,66_ = 3.65, *p* = .060, 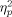 = .05, *β* = .50; Tab. 1).

### Preference for Novelty

As a Pearson correlation indicated a significant negative relationship between time spent moving in Trial 2 and novelty-preference (*r*_72_ = −.24, *p* = .043), this locomotor measure was used as a covariate in the analyses. While the main effect of sex was not significant (*F*_1,65_ = .29, *p* = .589), the main effect of age was significant (*F*_2,65_ = 3.59, *p* = .033, 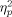 = .10, *β* = .65). However, an ANCOVA also showed a significant sex by age interaction in preference for novelty (*F*_2,65_ = 4.47, *p* = .015, 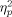 = .12, *β* = .75; Fig. 1c). Pairwise comparisons indicated that males exhibited greater novelty-preference than females at pnd 40 (*p* = .039), and females higher than males at pnd 80 (*p* = .049). There was no sex difference at pnd 28 (*p* = .320). Males showed an increase in novelty-preference from pnd 28 to pnd 40 (*p* = .043), with a non-significant decrease from pnd 40 to pnd 80 (*p* = .797), and no difference between pnds 28 and 80 (*p* = .439). Females exhibited no change in novelty-preference between pnd 28 and pnd 40 (*p* = 1.00), and a significantly higher novelty-preference at pnd 80 than at pnd 28 (*p* = .048) and pnd 40 (*p* = .013).

In order to check whether the total amount of time spent interacting with objects in Trials 1 or 2 influenced preference for novelty, we carried out additional analyses. Neither total object contact in Trial 1 nor total object contact in Trial 2 correlated with novelty preference (*r*s_72_ ≤ .08, *p*s ≥ .507). However, given that object contact significantly increased across the age groups, an additional ANCOVA was performed that also included object contact in Trial 1 and object contact in Trial 2 as covariates. The sex by age interaction remained significant (*F*_2,63_ = 3.82, *p* = .027, 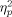 = .11, *β* = .68), the main effect of age difference was reduced (*F*_2,63_ = 2.50, *p* = .090), and the main effect of sex remained non-significant (*F*_2,63_ = .27, *p* = .607).

When all subjects were combined, a one sample *t*-test verified that the subjects exhibited a significant preference for novelty in Trial 2 (i.e., preference scores were greater than zero; *t*_71_ = 4.21, *p* < .001, *d* = .50, *β* = .99), with animals spending approximately 60% of contact time with the novel object and 40% with the familiar object. Males showed a preference for novelty at pnd 40 (*t*_11_ = 3.07, *p* = .011, *d* = .89, *β* = .80) but not at pnd 28 (*t*_11_ = 1.16, *p* = .272) or pnd 80 (*t*_11_ = 1.45, *p* = .175). Females exhibited a significant preference for the novel object at pnd 28 (*t*_11_ = 2.40, *p* = .035, *d* = .69, *β* = .59), and pnd 80 (*t*_11_ = 2.86, *p* = .015, *d* = .83, *β* = .74), but not pnd 40 (*t*_11_ = .37, *p* = .720).

### Preference Change

Although side biases were not apparent overall (*t*s_71_ ≤ .97 *p*s ≥ .337), there was an effect of trial by sex interaction on side biases (*F*_2,66_ = 5.23, *p* = .025, 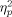 = .07, *β* = .62): females tended to show some changes in side bias between the trials (*p* = .061), whereas males did not (*p* = .190). The preference change measure takes into account side biases by comparing the proportion of time spent with each of the objects in Trial 1 with the proportion of time spent with the objects in Trial 2. Using locomotion as a covariate, the sex by age interaction was significant for preference change (*F*_2,65_ = 3.61, *p* = .033, 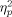 = .10, *β* = .65; Fig. 1d). The score was higher for males than females at pnd 40 (*p* = .019), but no longer at pnd 80 (*p* = .168). There were still no sex differences at pnd 28 (*p* = .930). Age differences in males remained with an increase between pnds 28 and 40 (*p* = .047), and a decreasing trend between pnds 40 and 80 (*p* = .072). There was still no difference in males between pnds 28 and 80 (*p* = 1.00). Females, however, no longer exhibited significant differences between any age groups (*p*s ≥ .345). *T*-tests revealed similar findings as before, except that, in females, preference for novelty was no longer significant at pnd 28 and only tended towards significance at pnd 80 (*t*_11_ = 2.10, *p* = .059, *d* = .61, *β* = .48). The main effects of age (*F*_2,65_ = 1.84, *p* = .167) and sex (*F*_1,65_ = .25, *p* = .617) were not significant.

## DISCUSSION

This study examined the ontogeny of response to novel objects in male and female Lister-hooded rats from adolescence to adulthood, using the NOR task with a short inter-trial interval. The results indicated that the strength of preference for the novel object in Trial 2 of the task exhibited a significant sex difference at mid-adolescence, with males showing a higher novelty-preference than females. This sex difference was not present at early adolescence, and, while the opposite pattern of results was observed at early adulthood, the adult sex difference was only present when calculated as preference for novelty, and not when calculated as preference change, suggesting that the adult sex difference is not robust. In contrast, other measures did not exhibit significant age by sex interactions, indicating that the sex difference in behavior at mid-adolescence was specific to situations involving choice of novelty. These results provide evidence that mid-adolescent rats exhibit a sex difference in behavior when provided with the opportunity to interact with a novel versus a familiar object that is not seen at younger or older ages.

Our finding that mid-adolescent male rats exhibit a stronger preference for novelty than females has not been reported previously. While two rodent studies have reported that the strength of the preference for the novel object in the NOR task peaks at adolescence (Calamandrei et al., 2002; Ricceri et al., 2000), neither reported a sex difference at this age despite testing subjects of both sexes. In both of these studies, sample sizes were smaller than in the current study (*n* = 4–5 per sex per age group, Ricceri et al., 2000; *n* = 8 per sex per age group, Calamandrei et al., 2002; *n* = 12 per sex per age group, current study). These previous studies also used mice rather than rats, and used a different methodology that involved multiple tests of object interactions in one experiment. Reger et al. (2009) failed to find an age difference in NOR performance in male rats, but used broad age classifications (pnd 29–40 for adolescents; pnd 50+ for adults) that could have masked more subtle age effects.

In our study, no sex differences were found in the total amount of object contact at any ages, and the analyses of co-variance confirmed that the sex difference in novelty-preference at mid-adolescence was robust to any differences in object contact or locomotor activity. Previously, we have reported that Lister-hooded rats do not exhibit sex differences in open field or elevated plus-maze behavior at mid-adolescence (Lynn & Brown, 2009, 2010), suggesting that the current results are not related to sex differences in anxiety-like responses at this age and are unique to a test that presents a “choice” of novel and familiar stimuli. The total object contact and locomotor activity gradually increased from early adolescence into adulthood, in support of previous research (e.g., Lynn & Brown, 2009, 2010; Moore, Linsenbardt, Melón, & Boehm, 2011; Renner, Bennett, & White, 1992) and potentially due to psychomotor development. The decrease in object interactions and locomotor activity between Trials 1 and 2, particularly in adolescence, could have resulted from habituation or from physical tiredness in subjects.

This study has shown that adolescent male rats exhibit a particularly strong preference for novelty during mid-adolescence compared both to females and to males at other ages. Interactions between the developing gonadal hormone system and dopamine neurotransmitter system could potentially underlie this finding. Adolescent rodents exhibit a higher vulnerability than adults to the positive rewarding properties of psycho-stimulants and other drugs of abuse (Doremus-Fitzwater, Varlinskaya, & Spear, 2010). Researchers have recently begun to examine how male and female adolescent rodents differ in their response to drugs of abuse (e.g., Hensleigh, Smedley, & Pritchard, 2011; Walker et al., 2009). Understanding sex and age differences in the response of rodents to natural rewards, such as novel objects, could enhance our understanding of age and sex differences in drug-misuse in humans.

## NOTES

This research was supported by the Wellcome Trust and the School of Psychology, University of St Andrews. We are grateful to Eric Bowman, Kevin Laland, and two anonymous reviewers for comments on the article.
